# microRNA-142–mediated repression of phosphodiesterase 3B critically regulates peripheral immune tolerance

**DOI:** 10.1172/JCI124725

**Published:** 2019-02-11

**Authors:** Nelomi Anandagoda, Joanna C.D. Willis, Arnulf Hertweck, Luke B. Roberts, Ian Jackson, M. Refik Gökmen, Richard G. Jenner, Jane K. Howard, Graham M. Lord

**Affiliations:** 1 School of Immunology and Microbial Sciences, King’s College London, London, United Kingdom.; 2 UCL Cancer Institute, University College London, London, United Kingdom.; 3 School of Life Course Sciences, King’s College London, London, United Kingdom.

**Keywords:** Autoimmunity, Immunology, Molecular genetics, T cells

## Abstract

Tregs play a fundamental role in immune tolerance via control of self-reactive effector T cells (Teffs). This function is dependent on maintenance of a high intracellular cAMP concentration. A number of microRNAs are implicated in the maintenance of Tregs. In this study, we demonstrate that peripheral immune tolerance is critically dependent on posttranscriptional repression of the cAMP-hydrolyzing enzyme phosphodiesterase-3b (*Pde3b*) by microRNA-142-5p (miR-142-5p). In this manner, miR-142-5p acts as an immunometabolic regulator of intracellular cAMP, controlling Treg suppressive function. *Mir142* was associated with a super enhancer bound by the Treg lineage–determining transcription factor forkhead box P3 (FOXP3), and Treg-specific deletion of miR-142 in mice (Treg^Δ142^) resulted in spontaneous, lethal, multisystem autoimmunity, despite preserved numbers of phenotypically normal Tregs. Pharmacological inhibition and genetic ablation of PDE3B prevented autoimmune disease and reversed the impaired suppressive function of Tregs in Treg^Δ142^ animals. These findings reveal a critical molecular switch, specifying Treg function through the modulation of a highly conserved, cell-intrinsic metabolic pathway. Modulation of this pathway has direct relevance to the pathogenesis and treatment of autoimmunity and cancer.

## Introduction

miRNAs are a class of small (~21–25 nucleotides), highly conserved, endogenous noncoding RNAs that regulate gene expression posttranscriptionally. This function is mediated through binding of the miRNA-containing RNA-induced silencing complex (miRISC) to complementary sequences in the 3′ UTR of target mRNAs, ultimately causing mRNA degradation or translational inhibition (1, 2). Through these regulatory actions, miRNAs have been shown to be critical for normal innate and adaptive immune processes (3), with aberrant expression implicated in multiple autoimmune diseases and malignancies (4, 5). Potential susceptibility to pharmacological manipulation with modified antisense oligonucleotides also makes them attractive therapeutic targets (6).

Tregs are primarily CD4 positive and play an essential role in maintenance of peripheral tolerance and avoidance of autoimmunity through the direct suppression of self-reactive effector T cells (Teffs). The Treg lineage is defined by expression of the transcription factor forkhead box P3 (FOXP3, also known as Scurfin), whose gene locus is located on the X chromosome (7). FOXP3 is exclusively expressed by Tregs and is critical for Treg lineage commitment. The importance of Tregs for maintenance of peripheral tolerance was highlighted by studies of the *Scurfy* mouse, which lacks functional Tregs due to a missense mutation in the murine *Foxp3* gene. These mice develop a severe lymphoproliferative disease with generalized multiorgan inflammation, leading to death by 24 days of age (8, 9). A similar outcome is observed in human patients suffering from immunodysregulation, polyendocrinopathy, enteropathy, X-linked (IPEX) syndrome. IPEX syndrome is also caused by mutations in the *FOXP3* gene and is characterized by defective Tregs, multisystem inflammation, and autoimmunity, with death usually by 2 years of age unless successfully treated (10, 11). Importantly, mice with a Treg-specific deletion of either Dicer (12–14) or Drosha (15), the 2 ribonuclease III (RNase III) enzymes necessary for the production and processing of mature miRNA species, also develop a spontaneous, lethal autoimmune disease virtually indistinguishable from that seen in *Scurfy* mice (7), demonstrating that miRNAs are critical for establishment of Treg-mediated peripheral tolerance. However, the set of miRNAs responsible for this functional deficiency has yet to be fully defined.

MicroRNA-142 (miR-142) is one of a handful of hematopoietic-specific miRNAs (16) and exists as 2 mature isoforms — miR-142-3p and miR-142-5p, generated by ribonuclease processing of the sense and antisense strands of the intact double-stranded miR-142 duplex. Of the 2 mature species, miR-142-5p is the predominant form expressed in thymically derived Tregs (17). Importantly, the mature sequence of miR-142 is evolutionarily conserved between murine and human species, making it an attractive target for translation from murine studies to human clinical use (18). miR-142-5p expression is downregulated in CD4^+^ T cells from patients with the multisystem autoimmune disease systemic lupus erythematosus (SLE) compared with healthy controls and overexpressed in an animal model of multiple sclerosis, suggesting that miR-142 plays a role in autoimmune disease (19, 20).

In this study, we show that miR-142-5p directly targets phosphodiesterase-3b (*Pde3b*) mRNA, limiting PDE3B protein levels in Tregs. PDE3B hydrolyzes its substrates cAMP and 3′-5′-cyclic guanine monophosphate (cGMP) to AMP and GMP, respectively, thus accelerating intracellular cAMP and cGMP turnover (21). The suppressive function of Tregs is critically dependent on the intracellular concentration of cAMP (22), with Tregs maintaining high levels of intracellular cAMP and Teffs requiring low cAMP levels to undergo activation (23). Collectively, our findings reveal a critical role for miR-142-5p in the regulation of intracellular cAMP concentration via modulation of *Pde3b* expression in Tregs and therefore place miR-142-5p in the center of the molecular circuitry that regulates Treg suppressive function.

## Results

### miR-142 is associated with a super-enhancer occupied by FOXP3 in Tregs.

**Figure 1:**
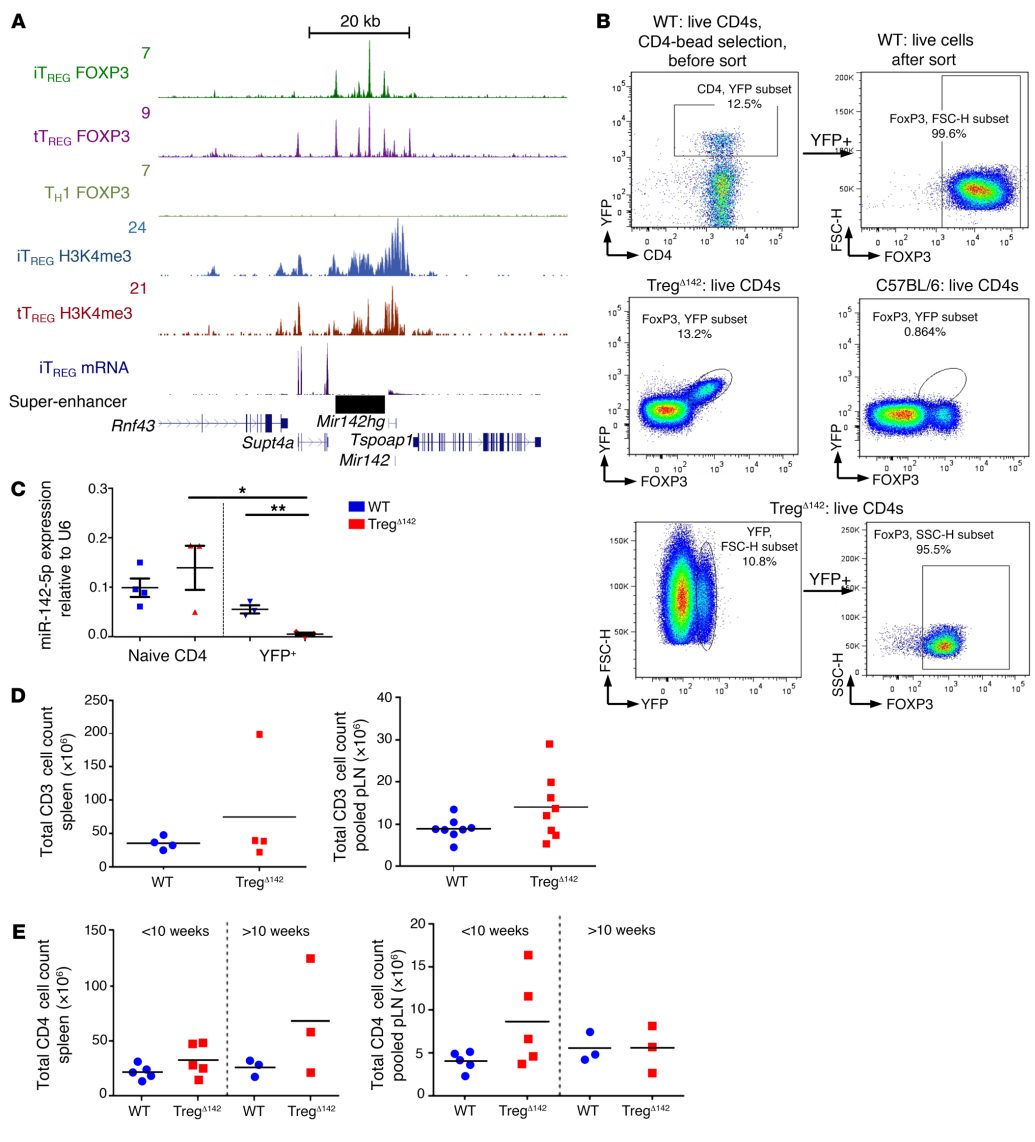
Treg^Δ142^ mouse: validation data. (**A**) ChIP-Seq binding profiles (reads/million, input subtracted) for FOXP3 and H3K4me3 and mRNA-Seq (reads/million) around *miR142* in Tregs. Genes and super-enhancers are shown below and a scale bar above. Tregs are defined as CD4^+^CD25^+^FOXP3^+^. (**B**) Flow cytometric gating on YFP (sorted and fixed live CD4^+^ cells) and concomitant/subsequent FOXP3 staining. (**C**) miR-142-5p expression in naive CD4^+^ T cells and YFP^+^ Tregs in WT and Treg^Δ142^ by RT-qPCR. *n* ≥ 3. *P < 0.05; **P < 0.001, 2-tailed Student’s *t* test. (**D**) Total CD3 counts in spleen (*n* = 4 per group) and peripheral lymph node. *n* = 7 per group. Nonsignificant, 2-tailed Student’s *t* test. (**E**) Total CD4 counts in spleen and peripheral lymph node in absence of disease (<10 weeks; *n* = 5; nonsignificant, 2-tailed Student’s *t* test) and presence of disease (>10 weeks; *n* = 3; nonsignificant, 2-tailed Student’s *t* test).

We first sought to identify miRNA genes related to the commitment to, or function of, the CD4^+^ Treg lineage. To address this, we utilized ChIP coupled with next-generation sequencing (ChIP-Seq) to identify whether any miRNA genes were associated with super-enhancers occupied by FOXP3, the lineage-determining transcription factor (LDTF) of Tregs. Foxp3 super-enhancers are genomic regions that exhibit particularly high occupancy of LDTF and transcriptional coactivators and tend to be associated with cell type–specific genes (24). The identification of super-enhancers has previously allowed for the definition of key lineage-specific genes critical for controlling T cell identity (24–26). Following our analysis, the only miRNA gene associated with a Foxp3 super-enhancer in Tregs was *Mir142*, located approximately 2 kb upstream of the fifth ranked Foxp3-associated super-enhancer (Figure 1A and Table 1). FOXP3 bound this locus both in thymically derived Tregs analyzed directly ex vivo (tTreg) and in Tregs induced in vitro from naive CD4^+^ T cells activated in the presence of TGF-β and IL-2 (iTreg) (Figure 1A). The *Mir142* locus was also associated with high levels of histone H3 lysine-4 tri-methylation (H3K4me3) in both tTreg and iTreg and was transcriptionally active (Figure 1A). These data suggest that miR-142 is important for Treg function.

### Generation and validation of Treg^Δ142^ mice.

We next set out to determine whether miR-142 was important for Treg biology or immune tolerance. To do this, we undertook a conditional gene-knockout strategy and generated a Treg-specific miR-142–deficient mouse (FoxP3^YFP–Cre^ × *Mir142^fl/fl^*; Treg^Δ142^, where YFP indicates yellow fluorescent protein). The FoxP3^YFP–Cre^ model allowed for deletion of a target gene in Tregs only, as previously described (27). Furthermore, because the FoxP3^YFP–Cre^ allele is X-linked, this allowed assessment of the effects of mosaic disruption of miR-142 in female mice that are heterozygous for FoxP3^YFP–Cre^ (FoxP3^YFP–Cre/WT^ × *Mir142^fl/fl^*). For clarity, Treg^Δ142^ refers only to male FoxP3 ^YFP–Cre^ × *Mir142^fl/fl^* and homozygous female FoxP3^YFP–Cre^ × *Mir142^fl/fl^* mice.

FoxP3 staining of YFP^+^ cells from these mice demonstrated that more than 95% of FACS-sorted YFP^+^ cells were FoxP3^+^ (Figure 1B), confirming the validity of YFP as a means of identifying Tregs in this model. Quantitative reverse-transcription PCR (qRT-PCR) conducted on total RNA isolated from YFP^+^ cells sorted from Treg^Δ142^ mice confirmed a complete absence of miR-142 transcripts, which were otherwise readily detectable in control cells, validating Cre-mediated deletion of the *Mir142* locus in Foxp3-expressing cells (Figure 1C). Non-Treg T cell populations from these mice demonstrated miR-142 expression levels comparable to those of controls, demonstrating a lack of detectable background Cre-recombinase activity in this model (Figure 1C). These mice also did not demonstrate any of the lymphopenic features previously reported in *Mir142^–/–^* animals (28), retaining a full spectrum of other T cell lineages with equivalent levels of miR-142 in naive CD4^+^ T cells from WT (FoxP3^YFP–Cre^ × *Mir-142^+/+^*), Treg^Δ142^, and WT YFP^+^ mice (Figure 1, D and E), further confirming Treg-specific deletion of miR-142 in Treg^Δ142^ mice.

### Treg^Δ142^ mice demonstrate a defect in Treg suppressive function despite apparently normal Treg lineage development.

**Figure 2:**
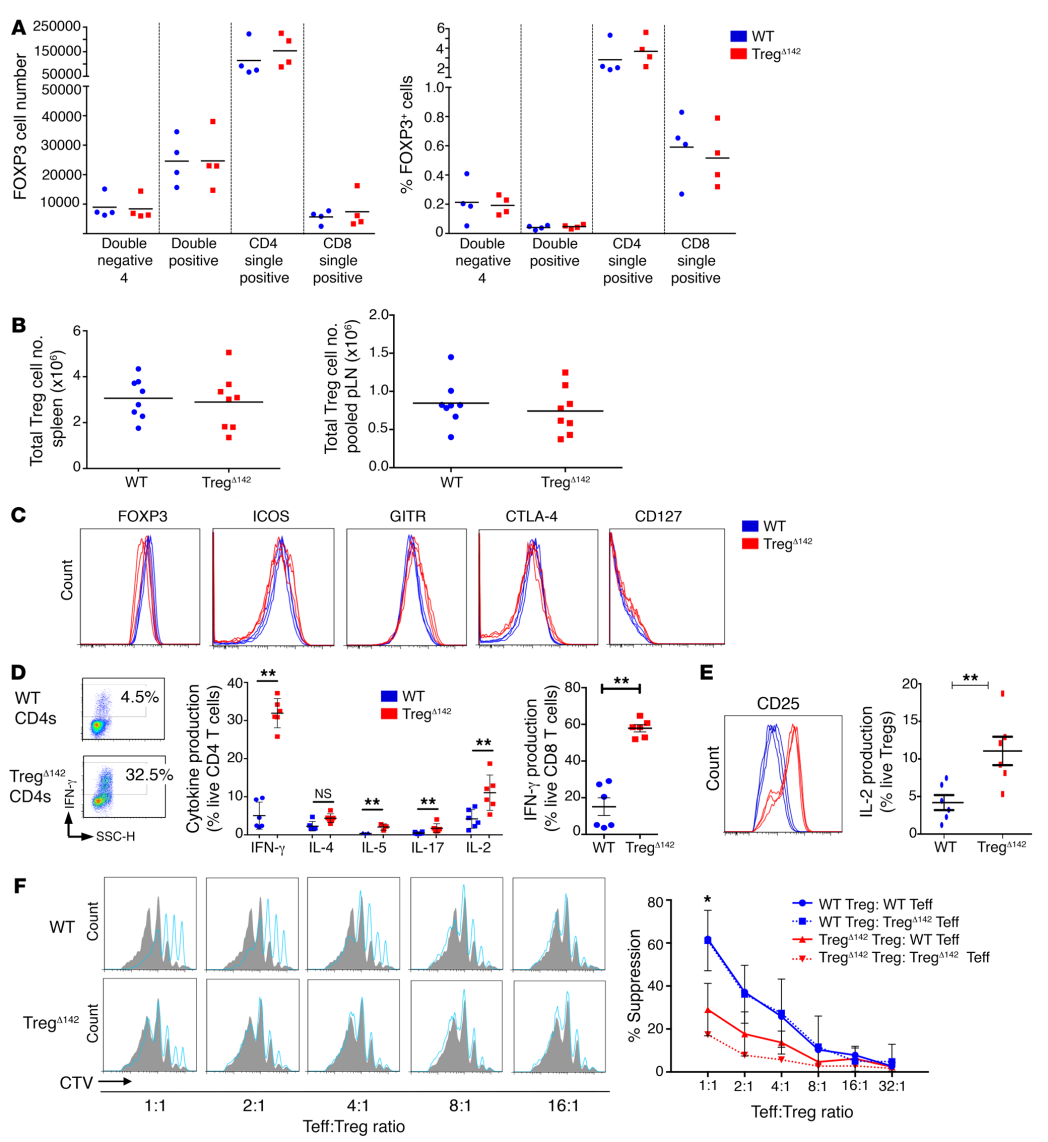
MiR-142–deficient Tregs develop normally, but fail to suppress Teff responses in vitro. (**A**) Number and proportion (%) of FOXP3^+^ (CD4^+^YFP^+^) cells at successive stages of thymus development (*n* = 4 per group). (**B**) Number of FOXP3^+^ cells in spleen (left) and peripheral lymph node (pLN) (right) samples (*n* = 8). (**C**) Flow cytometry histograms of peripheral Tregs (CD4^+^CD25^+^FOXP3^+^) stained for surface and intracellular Treg markers (*n* ≥ 4 per group). (**D**) Flow cytometry and cytokine secretion profiles of CD3^+^CD4^+^ and CD3^+^CD8^+^ T cells. **P < 0.01, 2-tailed Student’s *t* test. *n* ≥ 4 per group. (**E**) Flow cytometry and intracytoplasmic cytokine capture data for CD25 surface expression and IL-2 production in Treg^Δ142^ (CD4^+^YFP^+^) versus WT Tregs (CD4^+^CD25^+^FOXP3^+^). **P < 0.01, 2-tailed Student’s *t* test. *n* = 6 per group. (**F**) Coculture suppression assays; *P* value represents comparison of WT Tregs, WT Teffs, and Treg^Δ142^ WT Teffs (data combined from 3 independent experiments). CTV, CellTrace Violet. *P < 0.05, 2-tailed Student’s *t* test. *n* > 3 per group.

To establish the impact of Treg-specific miR-142 deletion on the development and biological function of Treg^Δ142^ Tregs, as well as the maintenance of peripheral immune tolerance, we examined the T cell compartment of the immune system of these animals in more detail. Immunological phenotyping of Treg^Δ142^ mice at 5 to 6 weeks of age revealed normal numbers of Tregs in the thymus (Figure 2A), spleen, and peripheral lymph nodes (Figure 2B). The expression of FOXP3 was unaffected in the absence of miR-142 (Figure 2C), and the canonical Treg markers ICOS, GITR, and CTLA-4 were equivalently expressed between peripheral Treg^Δ142^ and WT Tregs alongside the low expression of CD127 (IL-7Rα) typical of Tregs (Figure 2C). Intracytoplasmic cytokine staining of CD4^+^ cells isolated directly ex vivo from Treg^Δ142^ mice demonstrated an increased proportion of cells expressing cytokines, including IFN-γ, IL-2, IL-4, IL-5, and IL-17, versus WT CD4^+^ T cells*,* representing unchecked peripheral Teff responses. In addition, 60% of CD8^+^ T cells from Treg^Δ142^ mice produced IFN-γ, versus less than 20% of CD8^+^ T cells from WT mice (Figure 2D). Tregs from Treg^Δ142^ mice also demonstrated upregulated CD25 expression and IL-2 production (Figure 2E), which may reflect a compensatory increase in Treg activation in the absence of miR-142. Tregs purified from Treg^Δ142^ mice at 5 to 6 weeks of age completely failed to suppress Teff proliferation in an in vitro coculture suppression assay (Figure 2F), revealing a defect of Treg suppressive function despite apparently normal Treg lineage development and increased Treg activation. These data indicate that, following commitment to the Treg lineage, miR-142 is not essential for the maintenance of the peripheral Treg pool. However, its expression is critical for maintenance of the Treg activation state and optimal suppression of cytokine production by Teffs under steady-state conditions.

### The Treg^Δ142^ Treg suppressive defect is cell intrinsic and leads to development of lethal multisystem inflammatory disease.

**Figure 3:**
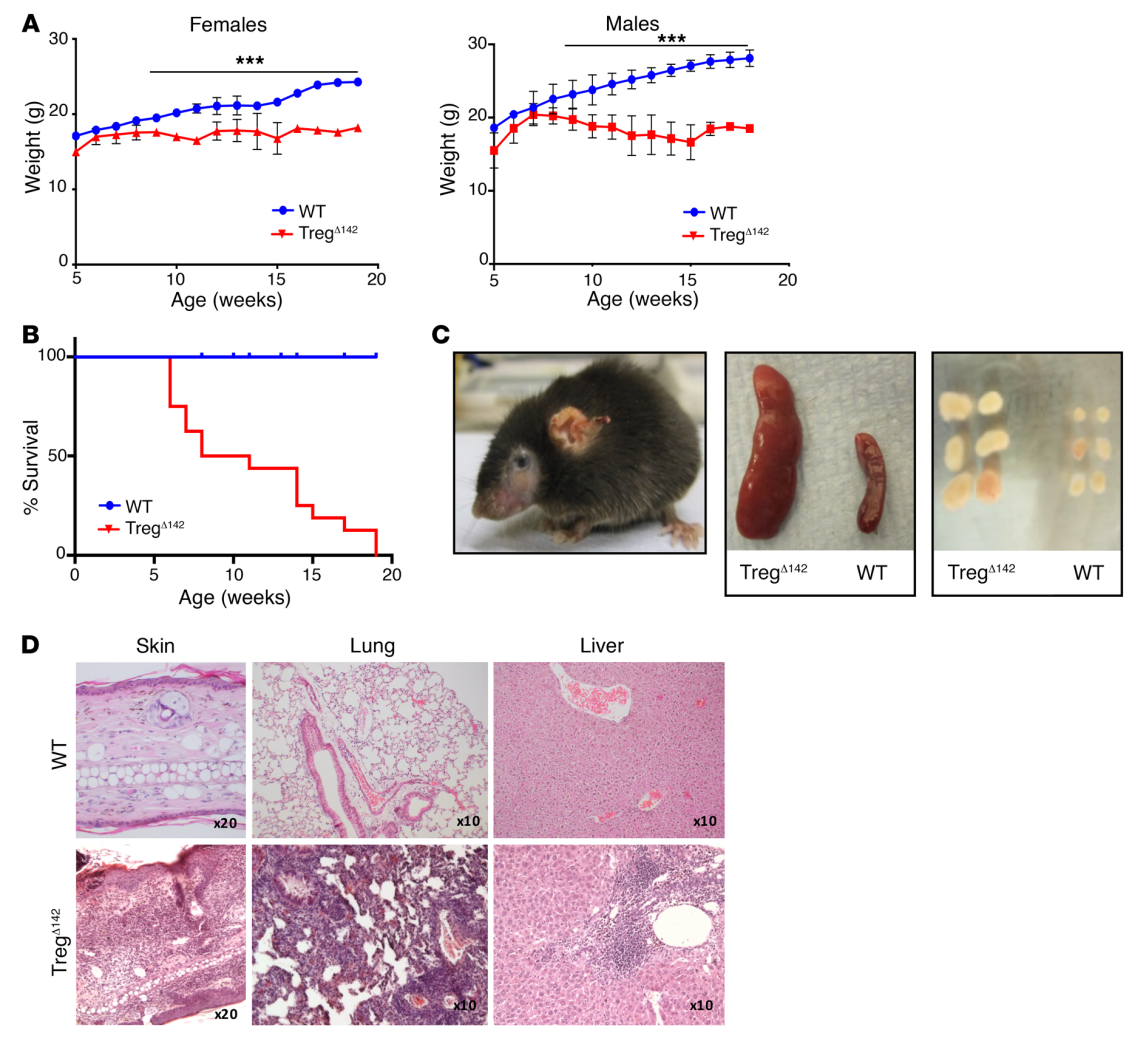
Treg-specific deficiency of miR-142 causes a multisystem lethal autoimmune syndrome due to a failure of peripheral tolerance. (**A**) Weight charts demonstrating weight loss from 7 weeks of age in Treg^Δ142^ males and females. ***P < 0.001, 2-tailed Student’s *t* test. *n* > 10 per group. WT and Treg^Δ142^ data are also shown in Figure 4A. (**B**) Survival of Treg^Δ142^ and WT littermate control mice. *P* < 0.001, 2-tailed Student’s *t* test. Data combined from 2 independent experiments. *n* > 10. (**C**) Scurfy-type phenotype seen in Treg^Δ142^ mice (16-week-old female mouse shown); gross splenomegaly and lymphadenopathy seen in Treg^Δ142^ mice. (**D**) H&E staining of formalin-fixed, paraffin-embedded sections from ear skin, liver, and lung. Original magnification, ×20 (skin); ×10 (lung, liver). WT and Treg^Δ142^ skin, lung, and liver histology are also shown in Figure 6E.

**Figure 4:**
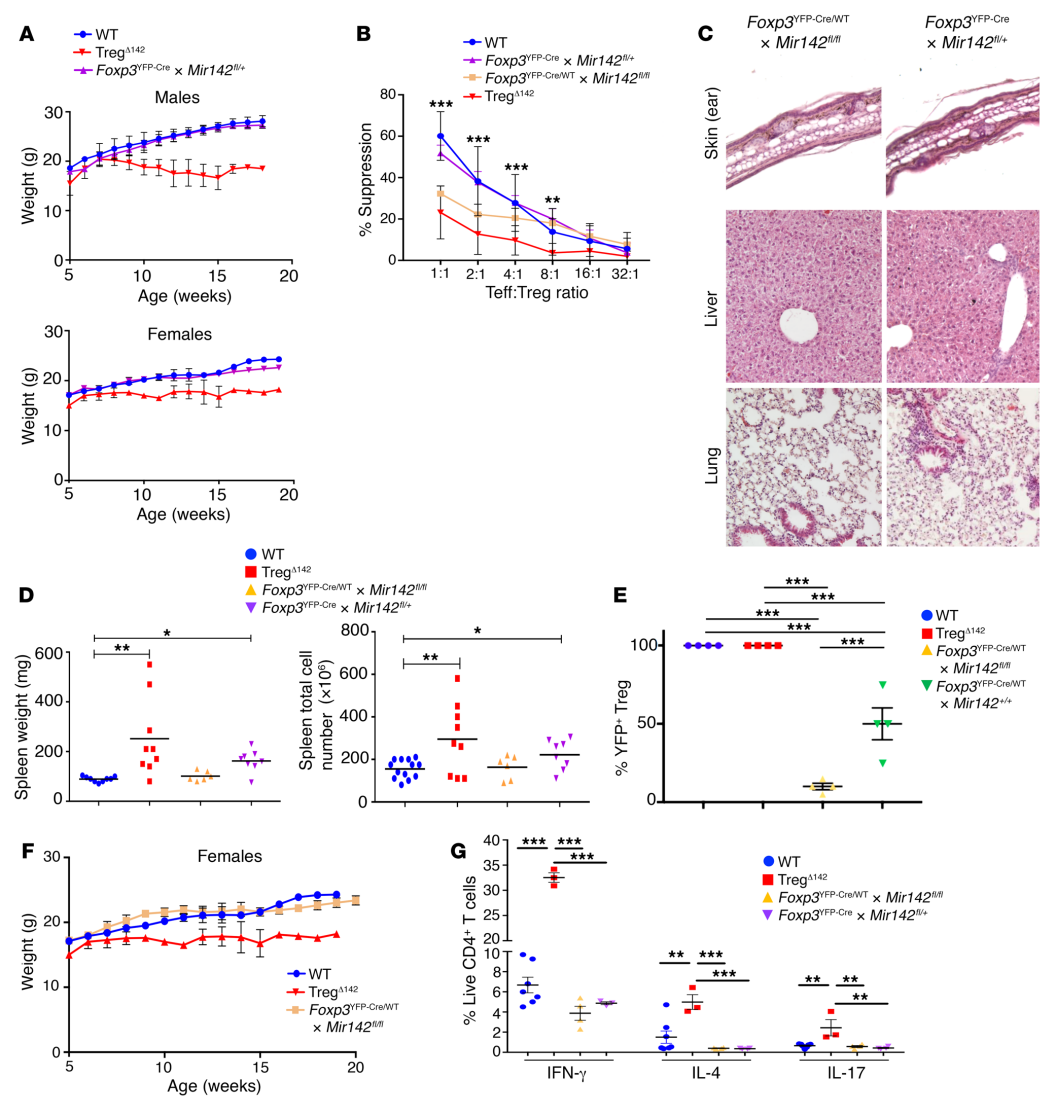
The cell-intrinsic Treg suppressive defect is directly attributable to cell-specific loss of miR-142 expression. (**A**) Weight charts of WT, Treg^Δ142^, and *Foxp3*^YFP–Cre^ × *Mir142^fl/+^* mice (male and female; *n* > 9). WT and Treg^Δ142^ data are also shown in Figure 3A. (**B**) Coculture suppression assays (data combined from 3 independent experiments). *P* values represent comparison between WT, Treg^Δ142^, *Foxp3*^YFP–Cre/WT^ × *Mir142^fl/fl^*, and *Foxp3*^YFP–Cre^ × *Mir142^fl/+^*. **P < 0.01; ***P < 0.001, 1-way ANOVA. *n* > 4 per group. No significant difference noted between WT and *Foxp3*^YFP–Cre^ × *Mir142^fl/+^*. (**C**) H&E staining of formalin-fixed, paraffin-embedded sections from ear skin, liver, and lung from *FoxP3*^YFP–Cre/WT^ × *Mir142^fl/fl^* and *Foxp3*^YFP–Cre^ × *Mir142^fl/+^* mice. Original magnification, ×10. (**D**) Comparison of spleen weights and cell counts. *P < 0.05; **P < 0.01, 1-way ANOVA. *n* > 6. (**E**) YFP^+^ Tregs as a percentage of the total Treg pool (CD4^+^CD25^+^FoxP3^+^) from WT, Treg^Δ142^, *Foxp3*^YFP–Cre/WT^ × *Mir142^fl/fl^* (female), and *Foxp3*^YFP–Cre^ × *Mir142^fl/+^* (male and female) mice. *n* = 4 per group. ***P < 0.001, 1-way ANOVA. (**F**) Weights of WT, Treg^Δ142^, and *Foxp3*^YFP–Cre/WT^ × *Mir142^fl/fl^* (female). *n* > 6 per group. One-way ANOVA. (**G**) Cytokine secretion profiles of CD3^+^CD4^+^ and CD3^+^CD8^+^ T cells from WT, Treg^Δ142^, *Foxp3*^YFP–Cre/WT^ × *Mir142^fl/fl^* (female), and *Foxp3*^YFP–Cre^ × *Mir142^fl/+^* mice (male and female). *n* ≥ 3. **P < 0.01; ***P < 0.001, 1-way ANOVA.

Given the functional suppressive defect displayed by Treg^Δ142^ Tregs, we anticipated that these animals may develop a *Scurfy-*like phenotype and, indeed, this was the case. Starting from 6 to 8 weeks of age, mice homozygous for the *Mir142^fl/fl^* allele, and possessing either 1 (male) or 2 (female) FoxP3^YFP–Cre^ alleles, developed a severe multisystem inflammatory disease, characterized by runting, weight loss, and death by 20 weeks (Figure 3, A and B). An early macroscopic feature of the disease phenotype was extensive dermatitis (Figure 3C). Postmortem examination was notable for marked lymphadenopathy and splenomegaly (Figure 3C). Histological examination of the liver, lungs, and skin of affected mice also revealed profound lymphohistiocytic infiltration (Figure 3D), very similar to that seen in the *Scurfy* mouse, as well as in mice lacking Treg-specific Dicer and Drosha (7, 12–15). In comparison with FoxP3^YFP–Cre^ mice homozygous for the floxed miR-142 allele, male and female FoxP3^YFP–Cre^ mice heterozygous for the floxed allele (*Mir142^fl/+^*) did not become terminally ill and remained visually healthy up to at least 12 months of age, with no weight loss or overt signs of disease (Figure 4A). Furthermore, Tregs from these mice retained normal suppressive activity in vitro (Figure 4B). However, histological examination of the liver, lungs, and skin revealed some mild/patchy inflammation (Figure 4C) accompanied by modest splenomegaly (Figure 4D). This indicated that, while for the most part, mice haplodeficient for miR-142 expression in Tregs were able to maintain Treg suppressive activity, full peripheral tolerance requires normal homozygous expression of miR-142, highlighting that the precise miR-142 gene dosage is critical for endowing full suppressive function.

Since the FoxP3^YFP–Cre^ allele is X linked, only 1 of the 2 FoxP3^YFP–Cre^ alleles is transcriptionally active in any given Treg from a female mouse heterozygous for FoxP3^YFP–Cre^ expression (FoxP3^YFP–Cre/WT^). Due to this random X inactivation, approximately 50% of Tregs in female FoxP3^YFP–Cre/WT^ mice should be predicted to express YFP (denoting FOXP3-Cre activity). However, while close to 50% of the total Treg pool from FoxP3^YFP–Cre/WT^ × *Mir142^+/+^* mice were found to be YFP^+^, in female FoxP3^YFP–Cre/WT^ animals possessing the floxed allele (FoxP3^YFP–Cre/WT^ × *Mir142^fl/fl^*), YFP^+^ cells (i.e., those in which the *Mir142* locus had been deleted) were found to represent less than 10% of the total Treg pool (Figure 4E). This observation implied a relative homeostatic defect of miR-142–deficient Tregs in the presence of miR-142–sufficient Tregs in vivo. This conclusion was further substantiated by the finding that female FoxP3^YFP–Cre/WT^ × *Mir142^fl/fl^* mice remained healthy up to at least 24 months of age, with no weight loss, overt disease, or histological evidence of inflammation (Figure 4, F and C). Similarly, cytokine secretion profiles of CD4^+^ T cells from these mice were comparable to those of littermate control WT mice, demonstrating that the predominance of miR-142–sufficient Tregs maintained immunological tolerance (Figure 4G). However, in comparison with YFP^neg^ Tregs, YFP^+^ Tregs from FoxP3^YFP–Cre/WT^ × *Mir142^fl/fl^* animals exhibited reduced in vitro suppressive activity of Teffs (Figure 4B), similar to the levels observed in YFP^+^ Tregs from Treg^Δ142^ mice. From this we infer that the miR-142–deficient Treg suppressive defect is cell intrinsic and directly attributable to cell-specific loss of miR-142 expression.

### Pde3b is a direct target of miR-142-5p in Tregs.

**Figure 5:**
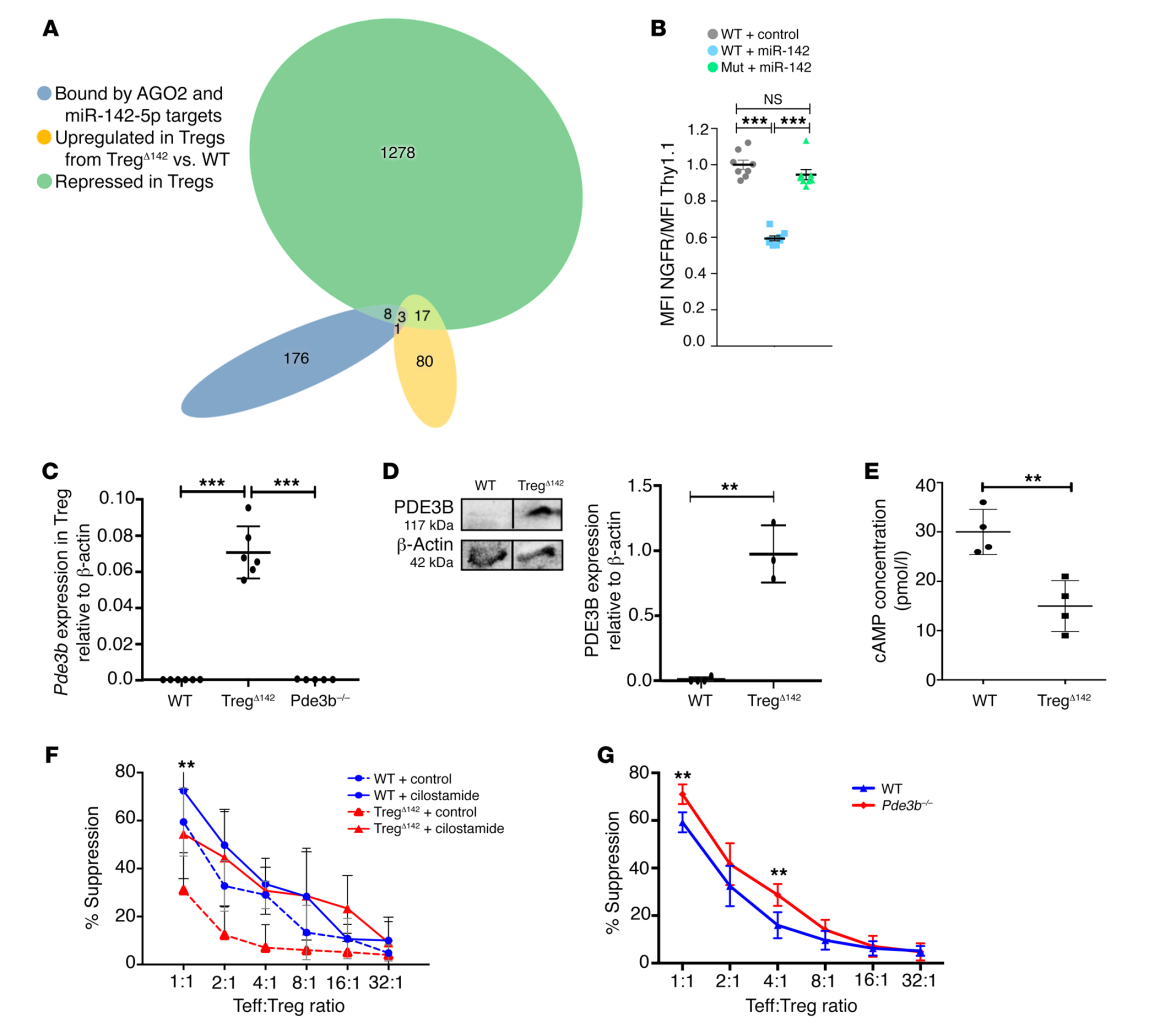
Identification of candidate miR-142 target genes in Tregs. (**A**) Intersection of genes harboring miR-142-5p–binding sites in their 3′ UTRs, as predicted by DIANA microT algorithm and by AGO2 HITS-CLIP in activated CD4^+^ T cells (29) (blue), with genes downregulated (≥2-fold, *P* < 0.05) in Tregs versus Teffs (30) (green) and genes upregulated in miR-142–deficient versus WT Tregs (≥2-fold, *P* < 0.05) (yellow). (**B**) Direct targeting of *Pde3b* mRNA 3′ UTR by miR-142. Expression of an NGFR reporter gene that has either the WT or mutated (Mut) *Pde3b* 3′ UTR miR-142-5p–binding site inserted in the presence of control or miR-142 expression vector in HEK293T cells. Data are representative of 2 independent experiments. Bars indicate mean and SD. *n* = 8. ***P < 0.001, Student’s *t* test. (**C**) *Pde3b* expression in Treg^Δ142^, *Pde3b^–/–^*, and WT Tregs by RT-qPCR. *n* = 6. ***P < 0.001, 2-tailed Student’s *t* test. Data from 2 independent experiments. (**D**) PDE3B expression in WT and Treg^Δ142^ Tregs by Western blot (relative density). *n* ≥ 3. **P < 0.01, 2-tailed Student’s *t* test. Lanes were run on the same gel but were noncontiguous. (**E**) cAMP ELISA data from Treg cell lysates. *n* = 4. **P < 0.001, 2-tailed Student’s *t* test. (**F**) Coculture suppression assay data comparing Treg suppressive function following in vitro cilostamide treatment of Tregs from WT and Treg^Δ142^ mice. *P* value signifies comparison between Treg^Δ142^ + cilostamide and Treg^Δ142^ + control. No significant difference between Treg^Δ142^ + cilostamide and WT samples. **P < 0.01, 1-way ANOVA. *n* ≥ 9 per group, combined from 3 independent experiments. (**G**) Coculture suppression assay data comparing Treg suppressive function in WT and *Pde3b^–/–^* mice. **P < 0.01, 1-way ANOVA. *n* ≥ 6 per group. Combined from 2 independent experiments.

To understand the mechanism underlying the phenotype associated with Treg-specific miR-142 deletion, we next attempted to identify genes directly regulated by miR-142 in Tregs. Argonaut 2 (AGO2) binds mature miRNAs as part of miRISC, which directs translational inhibition and degradation of target mRNAs (2). Therefore, to begin our target identification screen, we first identified mRNAs bound by AGO2 at predicted miR-142 target sites in activated CD4^+^ cells, utilizing publicly available AGO2 high-throughput sequencing of RNA isolated by crosslinking immunoprecipitation (HITS-CLIP) data (29). We further reasoned that Treg miR-142 target genes should be upregulated in Tregs in the absence of miR-142 and downregulated in WT Tregs versus Teffs. Thus, we required that candidate miR-142 target genes were upregulated (>2-fold, *P* < 0.05) in Treg^Δ142^ Tregs versus WT Tregs and downregulated (>2-fold, *P* < 0.05) in WT Tregs versus Teffs (30). As miR-142-3p is minimally expressed in Tregs compared with miR-142-45p (18), we reasoned that miR-142-5p was likely to be the main active mature miR-142 species in Tregs. Application of these stringent criteria identified 3 candidate miR-142-5p target genes: *Pde3b*, *Epas1*, and *Igf2bp3* (Figure 5A). To confirm that these genes were directly targeted by miR-142 as predicted, we utilized a flow cytometry–based reporter assay. The inclusion of the *Pde3b* 3′ UTR region containing the predicted binding site in the reporter construct led to robust repression of reporter gene expression in the presence of miR-142 (Figure 5B). This repression was completely reversed by mutation of the seed sequence at the predicted target site (Supplemental Figure 1B; supplemental material available online with this article; https://doi.org/10.1172/JCI124725DS1). In contrast, the repression was modest for the regions containing the predicted binding sites in the *Epas1* and *Igf2bp3* 3′ UTRs and was not relieved by mutating the seed sequences in either case (Supplemental Figure 2, A and B). Therefore, we concluded that *Pde3b* is a direct target of miR-142-5p and that the predicted miR-142-5p–binding sites in the 3′ UTRs of *Epas1* and *Igf2bp3* are not truly functional.

PDE3B regulates the intracellular concentration of cAMP and is critical for Treg function (23, 25). We therefore sought to determine whether the critical requirement of miR-142 for Treg suppressive function was associated with the direct repression of *Pde3b* by miR-142-5p. Supporting the hypothesis that miR-142-5p directly targets *Pde3b* in Tregs, qPCR confirmed significant overexpression of *Pde3b* in Treg^Δ142^ Tregs compared with controls observed by RNA-Seq (Figure 5C). However, *Pde3b* transcript levels in non-Treg T cell populations from Treg^Δ142^ and WT mice were not significantly different from one another (Supplemental Figure 3A), further validating the specificity of deletion of miR-142 in Treg^Δ142^ Tregs. In agreement with previous reports, we found that PDE3B protein was not detectable in WT resting Tregs (Supplemental Figure 3B) (23). However, PDE3B protein was clearly present in Treg^Δ142^ Tregs, as detected by Western blot (Figure 5D), congruent with findings of elevated *Pde3b* levels by qPCR. Consistent with increased PDE3B protein, intracellular cAMP levels in Treg^Δ142^ Treg lysate were lower compared with those in WT Treg control lysate (Figure 5E), indicating that, not only was there more PDE3B protein present in Treg^Δ142^ Tregs, but that the enzyme was also active. Importantly, the miR-142-5p target site in the Pde3b 3′ UTR is highly conserved among mammals (Table 2), supporting the likely translation of these findings in mice to human biology. Combined, these results show that *Pde3b* transcript levels are directly regulated by miR-142-5p in Tregs, leading to optimal Treg suppressive capacity through the maintenance of intracellular cAMP.

### Disruption of PDE3B activity through pharmacological inhibition or genetic ablation restores Treg^Δ142^ Treg–suppressive function and prevents lethal autoimmune disease.

If the suppressive capacity of Tregs from Treg^Δ142^ mice was impaired due to elevated expression of *Pde3b* in the absence of miR-142, inhibition of PDE3B would be predicted to reverse this effect. Cilostamide is a competitive inhibitor of PDE3A and PDE3B, but of the 2 isoforms, only PDE3B is present in T cells (22, 23). We found that pretreatment of Tregs from 5- to 6-week-old WT and Treg^Δ142^ mice with cilostamide (10 μM) prior to coculture with untreated Teffs in the absence of cilostamide restored the suppressive function of Treg^Δ142^ Tregs (Figure 5F). No rescue of suppressive function was observed when Teffs were pretreated with cilostamide (not shown). Tregs from *Pde3b*-deficient mice demonstrate marginal augmentation of ex vivo suppressive capacity when compared with WT Tregs, similar to that seen in WT Tregs treated with cilostamide in vitro (Figure 5, F and G). These data suggest that PDE3B may be expressed following TCR ligation in WT Tregs or that perhaps a low level of transient PDE3B expression may exist, consistent with previous reports (23). Importantly, while we observed some reduction of Treg viability in the absence of miR-142 during in vitro culture, mirroring the in vivo loss of competitive fitness in the presence of WT Tregs, the recovery of suppressive function following pharmacological inhibition of PDE3B was completely independent of this defect (Supplemental Figure 4). Thus, our data indicate that the defect in Treg function in the absence of miR-142 is independent of any observed homeostatic defect and is rescued by Treg-selective PDE3B inhibition.

**Figure 6:**
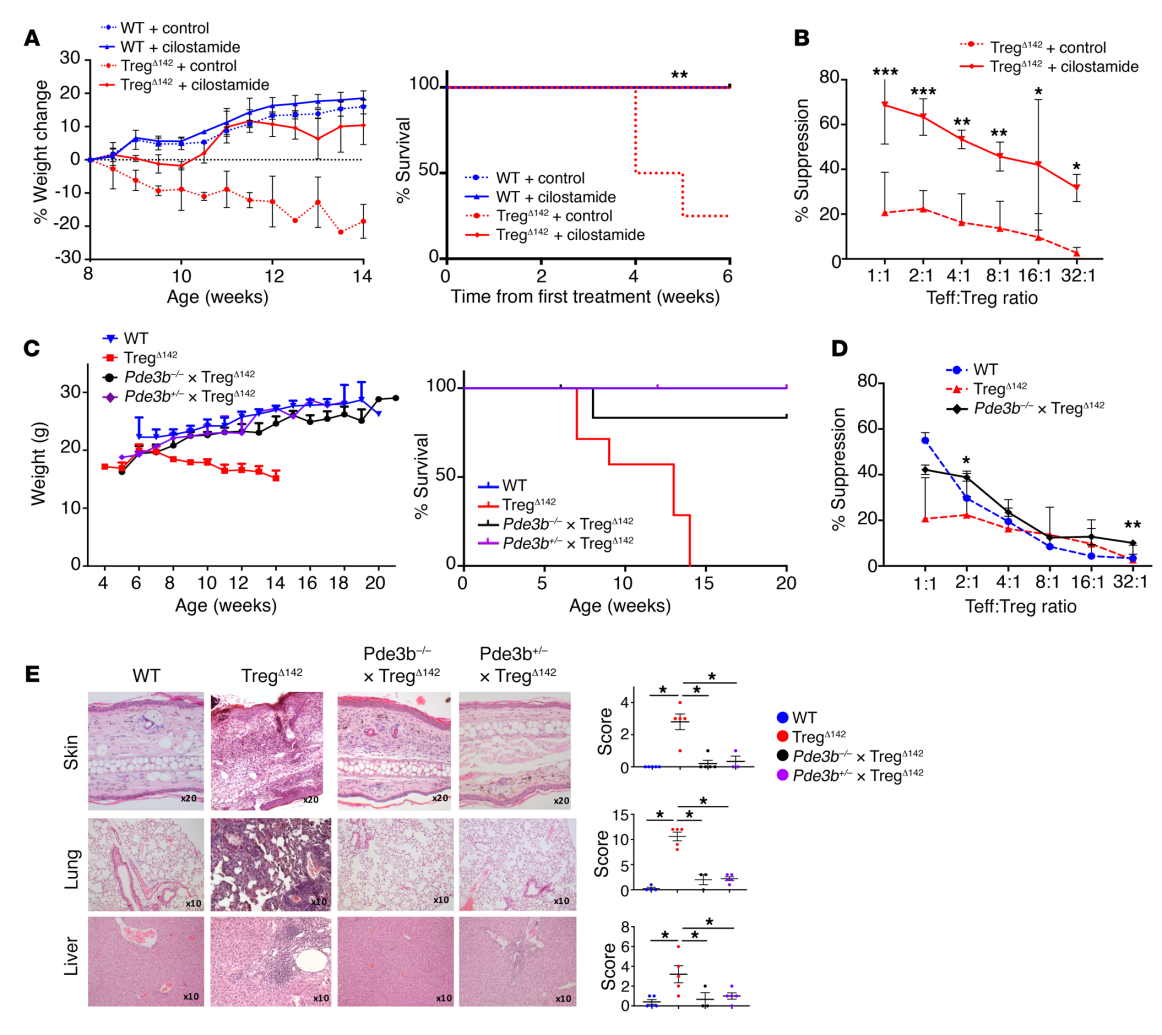
Pharmacological inhibition of PDE3B or genetic deletion of *Pde3b* reverses the lethality and phenotype of the autoimmune syndrome induced by Treg-specific loss of miR-142. (**A**) Weight (left) and survival (right) of Treg^Δ142^ and WT littermate control mice after 8 weeks of treatment with 6.4 mg/kg intraperitoneal cilostamide or control (*n* ≥ 3 for WT mice and *n* ≥ 6 for Treg^Δ142^ mice). Loss of more than 15% of body weight was the predefined mortality endpoint. (**B**) Coculture Treg suppression assay after 4 weeks of cilostamide treatment. *P < 0.05; **P < 0.01; ***P < 0.001, 1-way ANOVA. Data combined from 3 independent experiments. (**C**) Weight (left) and survival (right) of *Pde3b^–/–^* × Treg^Δ142^, Treg^Δ142^, Pde3b^+/–^ × Treg^Δ142^, and WT mice. *n* ≥ 5 (*Pde3b^–/–^* × Treg^Δ142^); *n* ≥ 3 (*Pde3b*^+/–^ × Treg^Δ142^); *n* ≥ 7 (Treg^Δ142^); and *n* ≥ 5 (WT mice). (**D**) Coculture Treg suppression assay comparing germline deletion of *Pde3b* (*Pde3b^–/–^* × Treg^Δ142^) with Treg^Δ142^ and WT littermate control mice. *P* values signify comparison between *Pde3b^–/–^* × Treg^Δ142^ and Treg^Δ142^. *P < 0.05; **P < 0.01, 1-way ANOVA. Data combined from 2 independent experiments. *n* ≥ 6. (**E**) H&E staining of formalin-fixed, paraffin-embedded sections from ear skin, liver, and lung (left) with histological scoring as described in Methods (right). Original magnification, ×20 (ear skin); ×10 (liver and lung). *P < 0.05, Student’s *t* test. *n* = 5 (*Pde3b^–/–^* × Treg^Δ142^, Treg^Δ142^ and WT); *n* = 3 (*Pde3b*^+/–^ × Treg^Δ142^). WT and Treg^Δ142^ skin, lung, and liver histology are also shown in Figure 3D.

We next sought to determine the relevance of this pathway in vivo. Remarkably, treatment of Treg^Δ142^ mice with cilostamide from 8 weeks of age prevented lethal autoimmune disease (Figure 6A), which was correlated with fully restored suppressive activity of miR-142–deficient Tregs (Figure 6B). To further validate that the defect observed in miR-142–deficient Tregs was a direct function of increased PDE3B levels, we generated a *Pde3b*-deficient, Treg-specific miR-142–deficient mouse (*Pde3b^–/–^* × FoxP3^YFP–Cre^ × *Mir142^fl/fl^*; *Pde3b^–/–^* × Treg^Δ142^). We found that these mice (in addition to littermates heterozygous for *Pde3b* germline deletion) remained healthy up to more than 20 weeks of age, with no weight loss, dermatitis, or overt disease, and exhibited restored ex vivo Treg suppressive function (Figure 6, C and D). Histological examination of the liver, lung, and skin showed evidence of only mild, patchy inflammatory infiltrate (Figure 6E). To exclude the possibility that this was due to impaired immunity in *Pde3b*-deficient mice, we examined their T cell effector function. *Pde3b^–/–^* mice had normal T cell numbers and effector function when compared with WT Teffs, as previously reported (23) (data not shown).

We therefore conclude that miR-142-5p represses *Pde3b* expression in Tregs and that this is essential for Treg suppressive function. In the absence of this critical molecular pathway, the mechanisms governing peripheral immune tolerance are compromised, resulting in a systemic lethal autoimmune syndrome.

## Discussion

The restriction of self-reactive peripheral Teff responses by Tregs is a core tenet of the mechanisms underlying peripheral tolerance, preventing development of autoimmunity. In the work presented here, we have demonstrated that miR-142 plays a critical, cell-autonomous role in facilitation of this Treg suppressive activity and, by virtue of this, successful orchestration of peripheral tolerance. By applying stringent target identification criteria and by validating these targets experimentally, the phosphodiesterase *Pde3b* was revealed to be a direct target of the miR-142 isoform miR-142-5p in Tregs. These data support the conclusion that, under WT conditions, direct repression of *Pde3b* by miR-142-5p is a key determinant of Treg function and peripheral immune tolerance.

The work presented in this study contributes to the understanding of the role of miRNAs in Treg biology. Previously, it has been shown that Tregs display a distinct miRNA expression profile when compared with conventional CD4^+^ Teffs (31), suggesting a role for specific miRNAs in the different functions of these cell types. Treg-specific miR-142 deletion did not appear to affect Treg lineage development in our study. However, iTreg development has been shown to be positively regulated by miRNAs, including miR-15b/16 (32), miR-99a (33), miR-126 (34), and miR-150, which target and suppress key components of the PI3K/AKT/MTOR signaling pathway, favoring Treg induction over Teff generation (35). Likewise, both iTreg and tTreg development are augmented by suppression of cytokine signaling 1 (SOCS1) through miR-155 (36). In contrast, other miRNAs, such as miR-17 (37) and miR-100 (38), can play inhibitory roles in Treg development via suppression of core components of the TGF-β–signaling pathway, including TGFβRII and SMAD2/3, or through direct suppression or destabilization of *Foxp3* mRNA, as has been shown for miR-10a (39), miR-15a/16 (40), miR-24 (41), miR-31 (42), miR-125a (43), miR-146a (44), and miR-210 (41). We revealed that miR-142-5p exerts its critical function in Tregs via facilitation of Treg suppression of Teffs. At the mechanistic level, this is achieved via maintenance of high Treg intracellular cAMP concentration, which is essential for subsequent transfer of cAMP to Teffs, suppressing their activation (21). In addition to transfer of cAMP, Tregs also suppress Teffs through CTLA4, limiting Teff-positive costimulatory signaling through CD28 interactions with CD80/86 in antigen-presenting cells. CTLA4 is reported to be targeted by both miR-15a/16 (40) and miR-145 (41) in Tregs; however, these interactions limit CTLA4 expression and reduce Treg suppressive function. Therefore, to our knowledge, this is the first report of an miRNA playing a direct, cell-intrinsic positive role in augmenting Treg suppressive activity.

Interestingly, while our study clearly demonstrates the critical requirement of miR-142-5p for maintenance of high intracellular concentration of cAMP in Tregs, miR-142-3p has previously been reported to restrict cAMP generation in CD4^+^ T cells through targeted suppression of adenylyl cyclase 9 (AC9) (45). Adenylyl cyclases are critically required for cAMP generation, and in their report, Huang et al. showed that Teffs maintain high levels of miR-142-3p in order to restrict AC9 expression, thus limiting endogenous cAMP production, which would otherwise inhibit their cellular activation (45). However, the authors reported that miR-142-3p expression is minimized in Tregs in order to support AC9 expression, facilitating cAMP generation so that Tregs may carry out suppression of Teffs. This is in line with other reports detailing miR-142-5p as the predominant miR-142 isoform found in Tregs (17). Our findings together with these data support a model whereby differential expression of miR-142 isoforms maintains Treg cAMP intracellular concentration. The synthesis of cAMP (low miR-142-3p, AC9 increased) as well as the inhibition of its hydrolysis to AMP (high miR-142-5p, PDE3B inhibited) may therefore represent parallel components of the same molecular goal.

Previously, it has been proposed that FOXP3 maintains the lineage stability and homeostasis of Tregs, in part by binding to and repressing *Pde3b* transcription directly, in order to maintain high levels of intracellular cAMP (23, 46). However, our data clearly demonstrate that in the absence of miR-142-5p, FOXP3-mediated repression of *Pde3b* is not sufficient to prevent significant upregulation and activity of PDE3B, leading to reduced intracellular cAMP and the breakdown of peripheral tolerance and lethality. Therefore, FOXP3 and miR-142-5p work in concert to maintain *Pde3b* repression, consistent with the established role of miRNAs in reinforcing transcriptional programs and conferring robustness to biological processes (47).

The worldwide surge in the incidence and prevalence of autoimmune disease over the last 30 years (including in younger people) is associated with a marked socioeconomic burden and has generated a global multibillion-dollar treatment market (48). Our findings represent a step forward in the understanding of the mechanisms governing peripheral immune tolerance, and conceivably, modulation of this pathway may provide a clinically tractable route for augmenting protective immune responses. Suppression of miR-142 expression in CD4^+^ T cells from patients suffering from autoimmune diseases such as SLE (19) predicts that *PDE3B* expression and activity may be enhanced in these cells and that this may underlie disease etiology. Modulation of this pathway, either through PDE3B inhibition or via exogenous manipulation of miR-142-5p expression levels or treatment with miRNA mimics, may therefore represent viable treatment options in the future. A number of PDE3 inhibitors are already widely used as therapeutic agents, for example, cilostazol in the treatment of intermittent claudication (49, 50). With over 100 clinical trials registered on ClinicalTrials.gov for cilostazol alone, the pharmacodynamic, tolerability, and safety profiles of these drugs have been under careful evaluation for a number of years. However, none of these trials is focused on assessing the potential therapeutic applications of PDE3 inhibitors in the treatment of autoimmune disease. In light of our findings, there is now a precedent for further investigation to establish whether modulation of PDE3B activity in Tregs may help to reestablish mechanisms of tolerance in patients suffering from autoimmune disease.

In addition to the potential relevance of our findings for treatment of autoimmune disease, identification of miR-142-5p as a critical metabolic regulator of Treg suppressive function may also have direct relevance to cancer immunotherapy, particularly when used for solid tumors. Tregs are enriched in cancer patients, particularly within and surrounding the tumor (51, 52). Furthermore, a large number of Tregs within tumors is frequently associated with poor clinical outcome for patients (53–56). The reasons for this are incompletely understood, but appear related to a tumor microenvironment rich in TGF-β (57), IL-10 (58), and adenosine (59), which promote Treg development, survival, and activity, resulting in enhanced suppression of tumor-infiltrating Teffs. Alongside other tactics employed by tumor cells, such as elevated expression of PD-L1, which promotes further suppression of antitumor Teff responses through inhibitory interactions with PD-1 expressed by activated Teffs (60), tumor-resident Tregs act to abolish antitumor T cell immunity through enhanced peripheral tolerance to or “immunological ignorance” of the tumor itself. Previously, tumor cell–specific miR-142-5p expression has been shown to promote tumor immune evasion through inhibition of phosphatase tensin homolog (PTEN), leading to downstream upregulation of PD-L1, restricting CD4^+^ T cell antitumor immunity (61), or inducing cancer stem cell–like properties. This in turn leads to enhanced growth and reduced apoptosis (62). However, the role of miR-142-5p specifically in human tumor-resident Tregs has yet to be addressed.

In summary, our results reveal that a critical function of miR-142-5p in Tregs is to facilitate Teff suppression via repression of *PDE3B*, leading to reduction of intracellular cAMP turnover. Therefore, this mechanism may prove a future avenue for Treg-targeted immunotherapies for autoimmune disease and solid tumors as well as for augmenting the response to pathogens.

## Methods

### Animals.

*Mir142^fl/fl^* mice were generated by homologous recombination in 129S mouse embryonic stem cells using a targeted vector containing both FRT and loxP sites flanking the *Mir142* locus as well as a neomycin resistance cassette to enable constitutive and conditional miR-142–deficient generation (performed by Genoway). Chimeric offspring were bred with C57BL/6J-Flp deleter mice to generate conditional lines, which were subsequently fully backcrossed onto a C57BL/6 background. Appropriate control mice were utilized in all experiments, with age- and sex-matched littermate FoxP3^YFP–Cre^ × *Mir142^+/+^* mice (WT) as controls for the FoxP3^YFP–Cre^ × *Mir142^fl/fl^* (Treg^Δ142)^ line. *Pde3b^–/–^* mice were a gift from Vincent Manganiello (NIH, Bethesda, Maryland, USA) and were generated as previously published (63). Chimeric offspring were bred with C57BL/6 mice to generate heterozygous lines, then bred with FoxP3^YFP–Cre^ × *Mir142^fl/fl^* and FoxP3^YFP–Cre^ × *Mir142^+/+^* mice to generate appropriate littermate controls to utilize in all experiments. The mice were housed under specific pathogen–free conditions.

### Flow cytometry and intracellular cytokine staining.

Single-cell suspensions were prepared from spleen and peripheral lymph nodes by tissue disruption and filtration. Following red cell lysis of splenocyte suspensions, an aliquot of 5 × 10^6^ splenocytes was stimulated with PMA at 1 ng/ml (MilliporeSigma) and ionomycin at 1 μg/ml (MilliporeSigma) for 4 hours at 37°C, 5% CO_2_, with the addition of monensin at 2 μM concentration (MilliporeSigma) for the last 2 hours. Stimulated and unstimulated samples were then Fc blocked and surface stained with fluorochrome-conjugated anti-mouse antibodies to LIVE/DEAD (Life Technologies) and combinations of CD45, CD3, CD4, CD8, CD25, CD44, CD62L, ICOS, GITR, CXCR3, and CD127 (eBioscience). A proportion of cells, including those stimulated with PMA and ionomycin, were fixed and permeabilized using a mouse Intracellular Staining Kit (eBioscience) per the protocol, and intracellular stains were then applied with fluorochrome-labeled anti-mouse antibodies to FoxP3, T-bet, CTLA-4, IFN-γ, and IL-17 (eBioscience). Appropriate single-stain controls were utilized for all fluorochromes. Cells were acquired on a Fortessa machine (BD Biosciences) and analyzed using FlowJo software (TreeStar).

For thymus flow cytometric analyses, thymocytes were harvested from 5- to 6-week-old mice, Fc blocked, and surface stained with fluorochrome-conjugated anti-mouse antibodies reporting LIVE/DEAD (Life Technologies). Half the cells were stained with a general panel consisting of anti-mouse antibodies to CD24, CD25, CD5, TCR-β, CD4, and CD8 (eBioscience), and the other half were stained with a double-negative panel consisting of anti-mouse antibodies to CD44, and CD25, and primary biotinylated antibodies to CD3, CD4, CD8, CD19, TCRγδ, CD11b, CD11c, Ly6G, NK1.1, and Ter119, with a subsequent secondary, fluorochrome-conjugated streptavidin step. All cells were fixed and permeabilized (as before), stained for Foxp3, and acquired and analyzed as before. For cells stained with the double-negative panel, dead cells and streptavidin-positive cells were excluded and the remaining cells were gated into successive double-negative populations by CD44 and CD25 (DN1 CD44^+^CD25^–^, DN2 CD44^+^CD25^+^, DN3 CD44^–^CD25^+^, DN4 CD44^–^CD25^–^). Full details for the antibodies used are listed in Supplemental Table 1.

### In vitro suppression assay.

CD4^+^ T cells were isolated from pooled peripheral lymph nodes and spleens of 5- to 6-week-old mice using CD4 microbeads (Miltenyi Biotec). Cells were labeled with fluorochrome-conjugated anti-mouse antibodies to CD4, CD62L, CD44 and CD25 (eBioscience) and sorted using a BD FACSAria II flow cytometric cell sorter (BD Biosciences) to more than 95% purity for YFP^+^CD4^+^ cells (Tregs) and naive (CD25^–^, CD62L^+^, CD44^–^) CD4^+^ T cells (Teffs). An aliquot of the sorted Treg population was stained with LIVE/DEAD and anti-CD25 antibodies, fixed, and permeabilized, then stained for FoxP3 and acquired on a flow cytometer (as before) to confirm purity. The Teffs were labeled with 10 μM CellTrace Violet (Life Technologies) according to the manufacturer’s instructions, washed, and then cultured in a 96-well U-bottom plate alone or with Tregs at ratios ranging from (Teff/Treg) 1:1 to 32:1, in triplicate, in the presence of anti-CD3 and anti-CD28 Dynabeads (Life Technologies), at a bead/cell ratio of 2:1, and RPMI 1640 cell culture medium (Gibco, Life Technologies) supplemented with 10% fetal calf serum, 50 μM 2-mercapto-ethanol, 2 μM l-glutamine, pyruvate, HEPES, nonessential amino acids, and antibiotics at 37°C 5% CO_2_. All 4 possible combinations of Tregs and Teffs from each group were utilized. After 72 hours in culture, the cells were stained with fluorochrome-conjugated anti-mouse antibodies to LIVE/DEAD (Life Technologies), and then proliferation of the Teffs was assessed by flow cytometry based on CellTrace Violet dilution (excluding YFP^+^ and dead cells). The numbers of nonproliferating cells (events in the first peak) and precursors of proliferating cells were calculated using standard formulae. Percentage suppression (*S*) of proliferation was calculated using the following formula: *S* = 100 − ([*c/d*] × 100), where *c* is the percentage of proliferating precursors in the presence of Tregs and *d* is the percentage of proliferating precursors in the absence of Tregs.

### Histology of tissue samples.

Mice were sacrificed between 6 and 20 weeks of age with age- and sex-matched WT controls. Samples of liver, lung, and ear skin were fixed in 10% neutral buffered formalin for 48 hours before paraffin embedding, sectioning, and staining with H&E (MilliporeSigma). Sections were scored blinded using histological scoring systems previously published for dermal inflammation (64) and lung/liver injury (65). Microscopy was performed with an Olympus BX51 microscope.

### Measurement of intracellular cAMP.

Tregs were isolated by flow cytometric cell sorting as above and washed 3 times with cold PBS. They were then lysed and the lysates used to measure cAMP using a Parameter cAMP Assay Kit according to the manufacturer’s instructions (R&D Systems).

### In vitro cilostamide treatment.

Tregs were isolated by flow cytometric cell sorting as above. They were then cultured in the presence of 10 μM cilostamide (Sigma-Aldrich) or equivalent PBS/DMSO control for 48 hours before being washed twice, counted, and used in an in vitro coculture suppression assay, as above. Pretreatment of Tregs with cilostamide prior to coculture, as opposed to treatment during the assay, was employed to prevent inadvertent treatment of Teffs as well as Tregs, excluding an effect of cilostamide on non-Treg populations in the suppression coculture assay.

### In vivo cilostamide treatment.

Littermate Treg^Δ142^ and WT mice were treated from 8 weeks of age with alternate day intraperitoneal injections of either 6.4 mg/kg cilostamide (MilliporeSigma) in PBS 10% DMSO or a control solution of PBS 10% DMSO. The mice were weighed every other day and monitored for signs of disease. A proportion of mice were sacrificed after 4 weeks of treatment, and Tregs were isolated directly ex vivo for use in an in vitro coculture suppression assay (as before). A proportion of mice were kept alive for as long as possible (either until they lost more than 15% of their body weight and had to be euthanized or until the end of the experiment).

### RNA extraction and RT-qPCR.

Total RNA was extracted using TRIsure (Bioline) according to the manufacturer’s instructions. MiR-142-5p RT-qPCR and cDNA reverse transcription were performed according to the manufacturer’s instructions using TaqMan assays (Applied Biosystems), with U6 small nuclear RNA or miR-191-5p endogenous controls. For *Pde3b* RT-qPCR, cDNA was prepared according to the manufacturer’s instructions using the Revertaid cDNA Kit (Thermo Fisher) and qPCR was performed using the Maxima Probe/ROX qPCR Master Mix (Thermo Fisher), with a specific primer/probe set for *Pde3b* (Life Technologies) and β-actin as an endogenous control. All qPCR reactions were analyzed using an ABI Prism 7900HT real-time PCR instrument (Applied Biosystems). Results were expressed relative to U6 or β-actin using the 2^−ΔCt^ method.

### FoxP3 ChIP-Seq in mouse Tregs.

CD4^+^ T cells from spleens and lymph nodes of 4- to 10-week old C57BL/6 mice were purified by CD4-positive selection (Miltenyi Biotec) followed by sorting of naive CD4^+^CD25^–^CD62L^hi^CD44^lo^ cells using a FACSAria II (BD Biosciences). Cells were activated by plate-bound anti-CD3 and anti-CD28 (both 10 μg/ml; clones 145-2C11 and 37.51, respectively; Bio X Cell). Tregs were generated by culturing in recombinant human TGF-β1 (33 ng/ml) and IL-2 (20 ng/ml, R&D Systems) for 7 days. Th1 cells were polarized as previously described (26). ChIP for FoxP3 was performed as described (26) using a mix of 2 antibodies: Santa Cruz sc-31738 and eBioscience FJK-16. Libraries were constructed and sequenced as previously described (26).

### Previously published sequencing data.

The following data sets were downloaded from the NCBI’s Gene Expression Omnibus database: mouse nTreg FoxP3 ChIP-Seq: GEO GSM999179 and GSM999181 (input) (66); mouse iTreg H3K4me3 ChIP-Seq: GEO GSM362005 (67); mouse nTreg H3K4me3 ChIP-Seq: GEO GSM362007 (67); and mouse iTreg_mRNA-Seq_: GEO GSM1480828 (25).

### ChIP-Seq and RNA-Seq data analysis.

Reads were filtered to remove adapters using fastq-mcf and for quality using seqkt. ChIP-Seq reads were aligned to mm9 with Bowtie2 (default settings). FOXP3 ChIP-Seq data were filtered for satellites and blacklisted regions (26) and super-enhancers identified with the ROSE algorithm using the default settings (68). RNA-Seq data were aligned with TopHat to mm9 (default settings) and converted to bigwig format as described (26). All ChIP-Seq data were deposited in the GEO database (GSE72279).

### Microarrays and target prediction algorithms.

Total RNA was extracted using TRIsure (Bioline) as before. cDNA was prepared using the Ovation Pico WTA Kit (NuGEN Technologies) and hybridized to Affymetrix Mouse Gene ST 2.0 microarrays by the King’s College London Genomics Centre facility. All cell populations used for the microarray analysis were generated in duplicate and individually processed. Raw data were processed with the robust multi-array average RMA algorithm (69) for probe-level normalization, and differential expression was estimated using the limma package in Bioconductor (70). Data from the arrays (GEO GSE122881) were compared with 2 public domain data sets: DIANA microT-predicted miR-142-5p target sites within CLIP-defined Ago2-binding sites in activated CD4^+^ T cells (29) and genes downregulated 2-fold or less at *P* = 0.05 in Tregs versus Teffs (30). Published microarray data were downloaded from GEO (GSE14350) and analyzed as described above.

### Development of flow cytometry–based miRNA-target reporter gene assay.

The direct interaction of an miRNA and a putative target is commonly confirmed by using a luciferase reporter assay in which the 3′ UTR of the gene tested is cloned downstream of a luciferase reporter gene. Using such an assay, we found that expression of miR-142 led to downregulation of the Renilla firefly reporter activity independently of the presence of the Pde3b 3′ UTR. These findings are consistent with a previous report (71) that identified several miR-142–binding sites in the Renilla luciferase cDNA. Therefore, we instead generated a new flow cytometry–based reporter system that contains the truncated human low-affinity nerve growth factor receptor (NGFR, also called CD271) gene as a readout for miRNA-target interaction (Supplemental Figure 1A). As an internal transfection control, the vector expresses the cell-surface marker Thy1.1 (also called CD90.1) under control of a separate promoter. Both expression cassettes were separated by a synthetic poly(A) signal and transcriptional pause element to stop read-through of the NGFR transcript.

The portion of *NGFR* encoding the intracellular domain was PCR amplified and inserted into BglII + EcoRI sites of pcDNA3 (Invitrogen). A second expression cassette consisting of the Thy1.1 cell-surface marker under the control of the phosphoglycerate kinase 1 promoter was inserted into NotI + SalI sites of pMY-IRES-EGFP (Cell Biolabs) from which the IRES-EGFP element had been removed. To avoid interference in expression between the 2 reporter genes, a synthetic poly(A) signal/transcriptional pause region was amplified by PCR using pGL4.13 (Promega) as a template, digested with XhoI/EagI, and inserted upstream of the PGK promoter into XhoI and NotI sites. A multiple cloning site consisting of restriction sites for EcoRI-NheI-SacII/NotI-XhoI was generated by annealing 2 synthesized oligonucleotides, which were ligated into EcoRI + XhoI sites. From this vector, the MCS-poly(A)-PGK-Thy1.1 cassette was excised with EcoRI + SalI and inserted into EcoRI + XhoI sites of pcDNA-tNGFR, resulting in pcDNA-tNGFR-poly(A)-PGK-Thy1.1 (Supplemental Figure 1A). A 207 bp fragment of the *Pde3b* 3′ UTR (base position 1448 to 1654) containing a predicted miR-142-5p target site was PCR amplified from cDNA and inserted into NotI + XhoI sites of pcDNA-tNGFR-poly(A)-PGK-Thy1.1. Reporter vectors contained positions 1–959 of the *Igf2bp3* 3′ UTR and positions 2253–2258 of the *Epas1* 3′ UTR. A mutated reporter construct was generated through substitution of 5 bases in the seed sequence (base position 1566 to 1570) by overlap PCR (Supplemental Figure 1B). Sequences of primers used are listed in Supplemental Table 2.

### miRNA-target reporter gene assay.

HEK293T cells were plated into 12-well plates at 7.3 × 10^4^ cells/well, 24 hours before transfection, and 3.28 ng pcDNA-tNGFR-poly(A)-PGK-Thy1.1 reporter plasmid containing either the WT or mutated *Pde3b* 3′ UTR and 1.17 μg pMY-miR-142-IRES-PAC or pMY-IRES-PAC control plasmid was cotransfected into each well using polyethyleneimine (Polysciences) in quadruplicate. Reporter expression was determined 48 hours after transfection by staining the cells with anti-NGFR-APC and anti-Thy1.1-eFluor450 (both eBiosciences), followed by staining with CYTOX Blue (Life Technologies) for LIVE/DEAD cell discrimination. Events were acquired with a BD Fortessa. Data are expressed as the ratio between the median fluorescence intensity (MFI) values obtained for NGFR and the Thy1.1 MFI values followed by averaging of the quadruplicate measurements. The reporter expression values from samples transfected with the miR-142 expression vector were normalized to the values of the samples transfected with the empty expression vector, which was set to 1.

### Western blot.

Cells were washed in ice-cold PBS and lysed in RIPA buffer (MilliporeSigma) according to the manufacturer’s instructions. The protein concentration was quantified using a Pierce BCA Protein Assay Kit (Thermo Scientific) according to the manufacturer’s instructions, and 20 μg protein was used for each sample. The lysed samples were then boiled in laemmli buffer and proteins resolved using SDS-PAGE (Bio-Rad) before being transferred to a nitrocellulose membrane. Blots were blocked with either 5% milk in Tris-buffered saline, 0.1% Tween-20 (TBST), or 5% BSA in TBS-Tween and then probed with rabbit anti-mouse Pde3b antibody (SMCP3B) (catalog NBPI-43333, Novus Biologicals). HRP-conjugated goat anti-rabbit IgG was used for secondary detection (GE Healthcare) and polyclonal β-actin antibody (catalog 4967, Cell Signaling Technology) used as an endogenous control. The blots were developed using enhanced chemiluminescence (Thermo Scientific/Pearce), images acquired using image lab 6.0.1 software, and the density of bands analyzed using Fiji ImageJ software (72).

### Statistics.

All between-group differences in flow cytometric, RT-PCR, microarray, and ELISA parameters were analyzed using 1-way ANOVA with Tukey’s test. Two-tailed Student’s *t* tests were used to analyze the flow cytometry–based reporter assay system results. Statistical analyses were carried out using GraphPad Prism 6 (GraphPad Software). *P* < 0.05 was considered statistically significant. Data are represented as mean ± SEM.

### Study approval.

All experiments were performed according to King’s College London and national guidelines, under a UK Home Office Project License (PPL:70/7869 to September 2018; P9720273E from September 2018).

## Author contributions

NA, JCDW, and GML conceived and designed the study. NA, JCDW, AH, MRG, IJ, and LBR acquired data. NA, JCDW, AH, MRG, and RGJ analyzed and interpreted data. NA, JCDW, AH, LBR, MRG, and IJ provided technical support. NA, JCDW, RGJ, JKH, and GML obtained funding. NA, LBR, and JCDW drafted the manuscript. GML supervised the study. AH, LBR, RGJ, JKH, and GML critically reviewed the manuscript.

## Supplementary Material

Supplemental data

## Figures and Tables

**Table 2:**
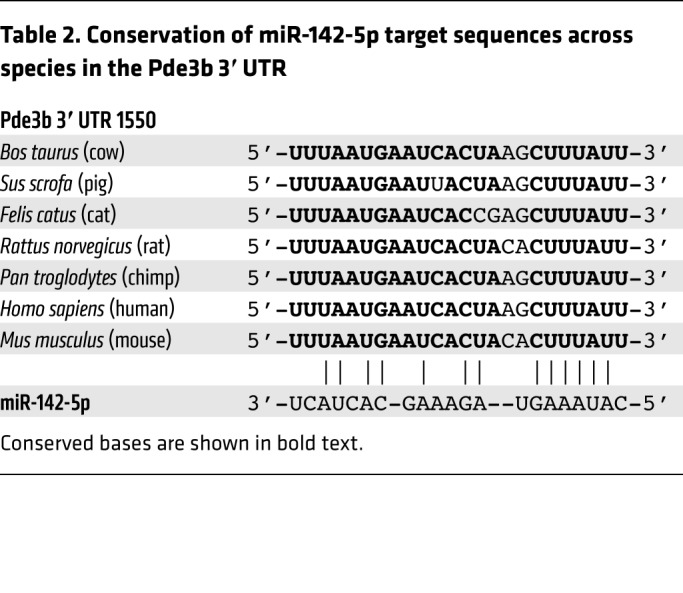
Conservation of miR-142-5p target sequences across species in the Pde3b 3′ UTR

**Table 1:**
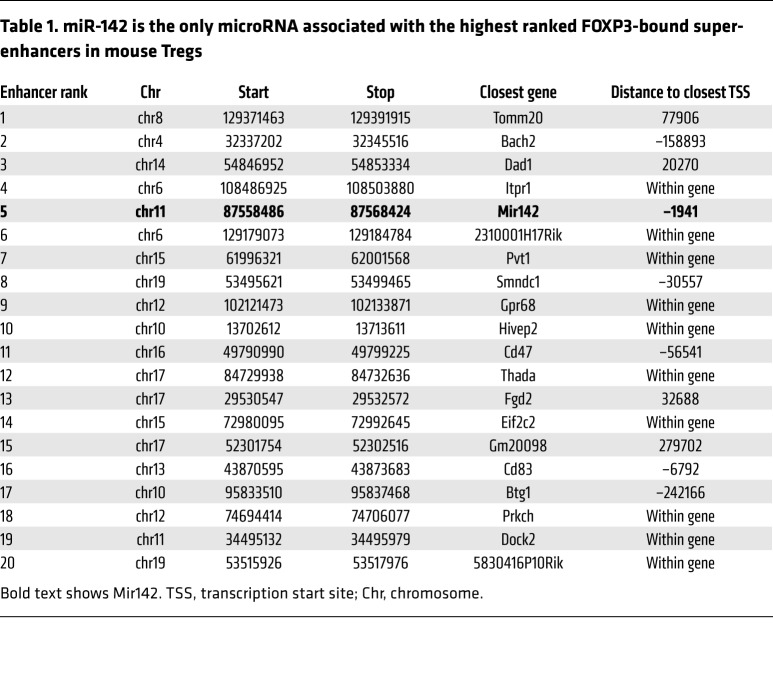
miR-142 is the only microRNA associated with the highest ranked FOXP3-bound super-enhancers in mouse Tregs

## Version 1 02/11/2019

Electronic publication

## Version 2 03/01/2019

Print issue publication
