# Supplementary material for: microRNA-142–mediated repression of phosphodiesterase 3B critically regulates peripheral immune tolerance
Source: J Clin Invest. 2019 Feb 11;129(3):1257–71. doi: 10.1172/JCI124725 (PMC6391082; doi:10.1172/JCI124725)
Supplement: Supplemental data [file jci-129-124725-s270.pdf]

A

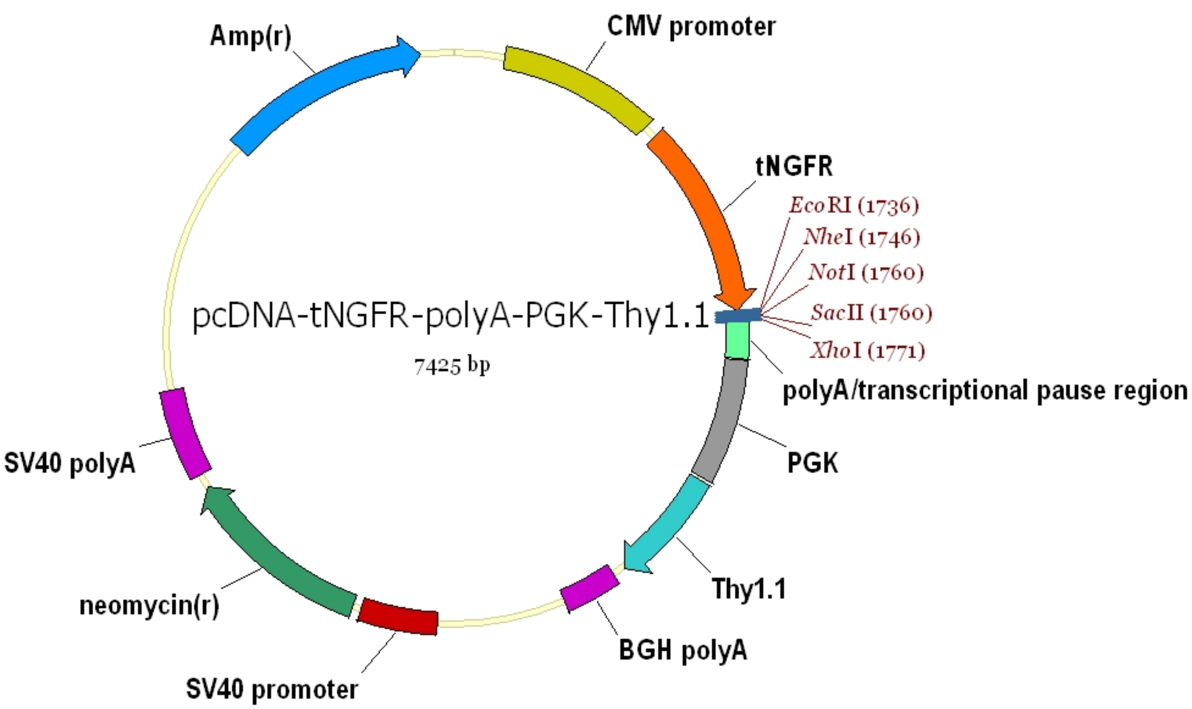

B

```
Original 3' UTR seed sequence:  5'-UUUAAUGAAUCACUACACUUUAUU-3'
                                     || ||  |  ||  |||||||
                  miR-142-5p:   3'-UCAUCAC-GAAAGA--UGAAAUAC-5'
                                     || ||  |  ||       ||
 Mutated 3' UTR seed sequence:  5'-UUUAAUGAAUCACUACUAGGGAUU-3'
```

Supplemental figure 1. The miR-target reporter gene flow-cytometry-based assay. (A) The reporter vector used to identify miRNA-target interactions. The vector expresses two cell surface molecules (tNGFR and Thy1.1) each under the control of a separate promoter. A poly(A) signal and transcriptional pause element serves to protect the Thy1.1-expression cassette from transcripts arising from the CMV promoter. 3'UTRs of transcripts tested are cloned into the multiple cloning site located downstream of the tNGFR gene. After co-transfection of the reporter vector with a miRNA-expression vector or a control vector, the expression levels of both cell surface markers are determined by flow-cytometry. Thy1.1 expression is used to normalise for differences in transfection efficiency between replicate wells **(B)** Mutation of the miR-142-5p seed sequence in the reporter construct: an overview of the interaction between miR-142-5p (middle) with the wild-type Pde3b 3'UTR (position 1550 to 1573 of Pde3b 3' UTR shown, top) and the target sequence containing five base substitutions in the seed sequence (bottom). Vertical lines indicate base pairing and mutated bases disrupting interaction between the miR-142-5p and the target sequence are highlighted in red.

A **B**

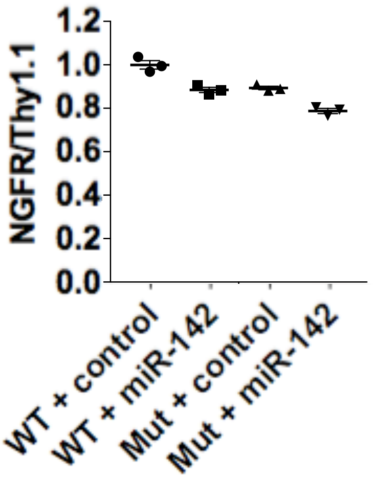

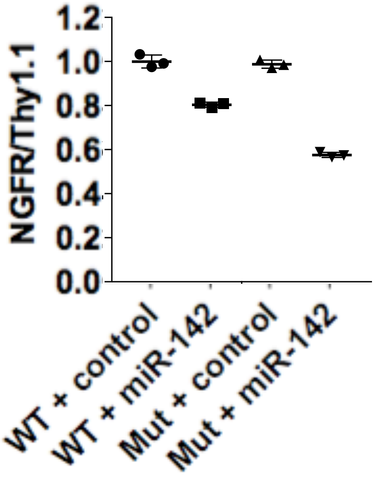

Supplemental figure 2. **Predicted miR-142-3p target sites in *Epas1* and *Igf2bp3* 3'UTRs are not functional in a miRNA reporter assay.** HEK293T cells were co-transfected with a dual reporter and miR-142 or control expression vectors. Reporter constructs contained regions of mouse 3'UTRs of *Igf2bp3* or *Epas1* encompassing predicted miR-142-3p sites (WT) or with mutated miR-142 seed sequence (Mut). NGFR reporter expression was measured 48 hours after transfection and normalized to Thy1.1 expression. Values are relative to normalized reporter expression in control transfected cells. Data represent one experiment and values are means ± SEM from three independent transfections. **(A)** *Igf2bp3* 3'UTR **(B)** *Epas1* 3'UTR

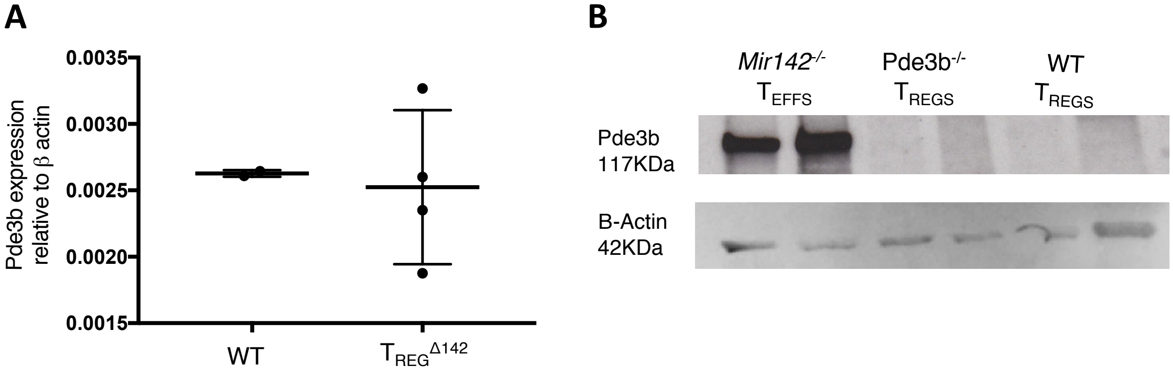

Supplemental figure 3. (A) *Pde3b* expression in $T_{REG}^{\Delta 142}$ and WT non-T_{REG} T cell by RTqPCR. (n = 4; non significant, two-tailed Student's t test) **(B)** *Pde3b* expression in *Mir142*$^{-/-}$ T_{EFFS} (positive control), Pde3b$^{-/-}$ T_{REGS} (negative control) and WT T_{REGS} by Western blot.

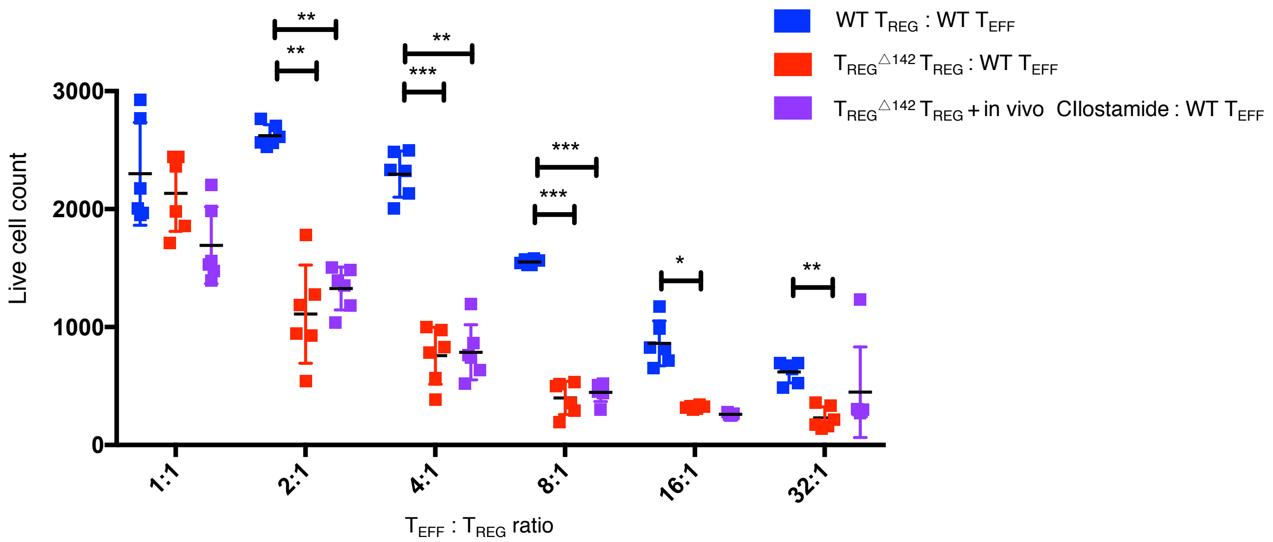

Supplemental figure 4. T_{REG} viability. Live cell count after 72 hours in vitro co-culture T_{REG} suppression assay ($n \geq 6$; $*p<0.05$, $**p<0.01$, $***p<0.001$, Student's t-test).

Antibody	Source	Catalogue Number / Clone
Live Dead Yellow	Thermo Fisher Scientific	L34959
CD45 Alexa Fluor 700	Thermo Fisher Scientific	56-0451-82 / 30-F11
CD3e PE	Thermo Fisher Scientific	12-0031-83 / 145-2C11
CD3e Pacific Blue	Thermo Fisher Scientific	HM3428 / 500A2
CD4 PE	Thermo Fisher Scientific	12-0041-82 / GK1.5
CD4 PerCPCy5.5	Thermo Fisher Scientific	45-0042-82 / RM4-5
CD8 APC Cy7	Thermo Fisher Scientific	A15386 / 53-6.7
CD8 PE	Thermo Fisher Scientific	MA1-10304 / 53-6.7
CD25 AlexaFluor 488	Thermo Fisher Scientific	53-0251-82/ PC61.5
CD25 APC-Cy7	Thermo Fisher Scientific	17-0251-82 / PC61.5
CD25 PE	Thermo Fisher Scientific	12-0251-82 / PC61.5
CD44 PECy7	Thermo Fisher Scientific	25-0441-82 / IM7
CD44 Pacific Blue	Thermo Fisher Scientific	48-0441-82 / IM7
CD62L Pacific Blue	Thermo Fisher Scientific	RM4328 / MEL-14
CD62L PE	Thermo Fisher Scientific	12-0621-82 / MEL-14
ICOS PECy5	Thermo Fisher Scientific	15-9942-81 / 7E.17G9
GITR PECy7	Thermo Fisher Scientific	25-5872-82 / DTA-1
CXCR3 PE	Thermo Fisher Scientific	12-1831-82 / CXCR3-173
CD127 APC	Thermo Fisher Scientific	17-1271-82 / A7R34
FoxP3 Alexa Fluor 700	Thermo Fisher Scientific	56-5773-82 / FJK-16s
FoxP3 PECy7	Thermo Fisher Scientific	25-5773-82 / FJK-16s
FoxP3 APC	Thermo Fisher Scientific	17-5773-82 / FJK-16s
T-bet PE	Thermo Fisher Scientific	12-5825-82 / 4B10
CTLA-4 APC	Thermo Fisher Scientific	17-1522-82 / UC10-4B9
IFNγ PE	Thermo Fisher Scientific	12-7311-82 / XMG1.2
IFNγ Pacific Blue	Thermo Fisher Scientific	48-7311-82 / XMG1.2
IL-17A AlexaFluor 488	Thermo Fisher Scientific	53--7177-82 / eBio17B7
IL-17A PECy7	Thermo Fisher Scientific	25-7177-82 / eBio17B7
IL-2 APC	Thermo Fisher Scientific	17-7021-82 / JES6-5H4
IL-4 FITC	Thermo Fisher Scientific	11-7042-82 / BVD6-24G2
IL-4 PE	Thermo Fisher Scientific	12-7042-82 / BVD6-24G2
IL-5 PE	Thermo Fisher Scientific	12-7052-82 / TRFK5
IL-10 Alexa Fluor 700	Thermo Fisher Scientific	56-7101-82 / JES5-16E3
CD3 Biotin	Thermo Fisher Scientific	13-0037-82 / OKT3
CD4 Biotin	Thermo Fisher Scientific	36-0041-85 / GK1.5
CD8 Biotin	Thermo Fisher Scientific	13-0081-85 / 53-6.7
CD19 Biotin	Thermo Fisher Scientific	13-0193-82 / 1D3
TCRγδ Biotin	Thermo Fisher Scientific	13-5711-82 / GL-3
CD11b Biotin	Thermo Fisher Scientific	13-0112-82 / M1/70
CD11c Biotin	Thermo Fisher Scientific	13-0114-82 / N418
Ly6G Biotin	Thermo Fisher Scientific	13-5931-82 / RB6-8C5
TER-119 Biotin	Thermo Fisher Scientific	MA5-17819 / TER119
NK1.1 Biotin	Thermo Fisher Scientific	13-5941-82 / PK136
Streptavidin	Thermo Fisher Scientific	45-4317-82

Supplemental table 1. Antibodies used in flow cytometry and intracellular cytokine staining

Primer name	Primer sequence
Pde3b 3'UTR F	CATGCGGCCGCGATGCTGGAATTTCTTACCTACCTAA
Pde3b 3'UTR R	GATCTCGAGTATGGTGGGACCAGTTTACAAATG
miR-142-5p seed mutation sense	AATGAATCACTACTA**GGGAT**TTATTAAACAT
miR-142-5p seed mutation antisense	ATGTTTAATAA**ATCCC**TAGTAGTGATTCATT
poly(A)/transcriptional pause F	TGACTCGAGAATAAAATATCTTTATTTTCATTACATCTG
poly(A)/transcriptional pause R	CGACGGCCGAGAGAAATGTTCTGGCACCTGC
tNGFR F	CGTAGATCT*GCCACC*ATGGACGGGCCGCGCCTGCT
tNGFR R	CTGGAATTCCTAGAGGATCCCCCTGTTCCACCTCT
MCS for reporter vector sense	AATTCTCACGCTAGCTCTAGCCGCGGCCGCATCAC
MCS for reporter vector antisense	TCGAGTGATGCGGCCGCGGCTAGAGCTAGCGTGAG
PGK-Thy1.1	TCAGCGGCCGCAATTCTACCGGGTAGGGGAGGC
PGK-Thy1.1	GCTGTCGACTCACAGAGAAATGAAGTCCAGGGCTTG

Supplemental table 2. Primer sequences used in cloning of the miR-target reporter vector. Restriction sites are underlined, the Kozac sequence is indicated in italics and bases mutated in the miR-142-5p seed sequence are highlighted in bold.
